# Poster Session I - A155 RUPTURE AT THE SHORELINE: FLOOD SYNDROME WITH SPONTANEOUS EVISCERATION

**DOI:** 10.1093/jcag/gwaf042.155

**Published:** 2026-02-13

**Authors:** R Ahmed, P Kundapur

**Affiliations:** Medicine, University of Saskatchewan, Saskatoon, SK, Canada; Medicine, University of Saskatchewan, Saskatoon, SK, Canada

## Abstract

**Background:**

Flood syndrome is the spontaneous rupture of an umbilical hernia with acute ascitic leakage and is a rare but life-threatening complication in cirrhotic patients, with mortality rates of 30–50%. Umbilical hernias occur in up to 20% of cirrhotic patients with ascites. Severe sequelae such as bowel evisceration are rare and sparsely described. The condition’s rarity and high perioperative risk create uncertainty in management.

**Aims:**

This case highlights Flood syndrome complicated by spontaneous bowel evisceration.

**Methods:**

case report

**Results:**

A 54-year-old man with alcohol-related cirrhosis presented with spontaneous umbilical ascitic leak. He denied trauma but reported ongoing alcohol use. Examination revealed an ulcerated umbilical hernia with a 2-cm defect. Surgical team recommended non-operative management due to high operative risk and patient preference. Labs showed INR 2.2, Bilirubin 104 μmol/L, Albumin 20 g/L, and Creatinine 499 μmol/L (baseline 70 μmol/L). Nephrotoxins were held, and he received 25% albumin 100 mL IV TID with broad-spectrum antibiotics. AKI limited further ascites management. Ongoing alcohol use rendered him ineligible for transplant. A TIPS work-up was initiated; his MELD score (>18) indicated a high risk of short-term mortality. Within 48 hours, he developed spontaneous bowel evisceration through the umbilical defect, requiring emergent surgical reduction, JP drain, and ostomy appliance for drainage.

**Conclusions:**

Flood syndrome results from rupture of an umbilical hernia under chronic intra-abdominal pressure due to ascites and portal hypertension. Complications include auto-paracentesis, cellulitis, peritonitis, and rarely evisceration. Conservative care carries high morbidity and mortality, while elective repair after optimization lowers mortality (≈6–20% vs. 60–80%). Non-cirrhotic hernias >1 cm is typically repaired electively, but cirrhotics face higher perioperative risk, historically leading to delayed intervention. However, studies show elective repair after medical optimization has better outcomes than emergent repair. No literature correlates umbilical defect size with evisceration risk. In this case, evisceration occurred through a 2-cm defect within 48 hours, suggesting even small defects may predispose to bowel evisceration. Ongoing management should be guided by a multidisciplinary team including Hepatology, Surgery, and Anesthesia.

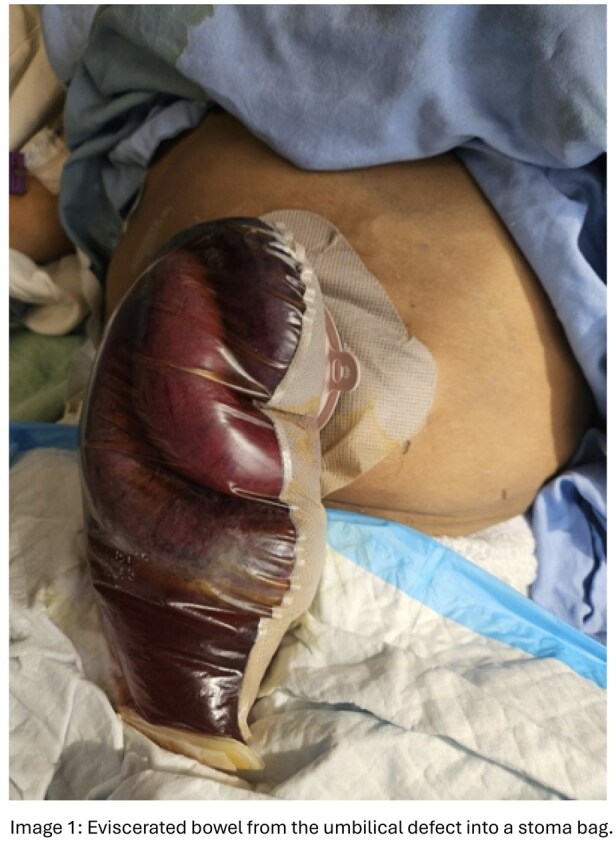

**Funding Agencies:**

None

